# RNA Interference Can Rebalance the Nitrogen Sink of Maize Seeds without Losing Hard Endosperm

**DOI:** 10.1371/journal.pone.0032850

**Published:** 2012-02-29

**Authors:** Yongrui Wu, Joachim Messing

**Affiliations:** Waksman Institute of Microbiology, Rutgers University, Piscataway, New Jersey, United States of America; University of Guelph, Canada

## Abstract

**Background:**

One of the goals of plant breeding is to create crops to provide better nutrition for humans and livestock. Insufficient intake of protein is one of the most severe factors affecting the growth and development of children in developing countries. More than a century ago, in 1896, Hopkins initiated the well-known Illinois long-term selection for maize seed protein concentration, yielding four protein strains. By continuously accumulating QTLs, Illinois High Protein (IHP) reached a protein level 2.5-fold higher than normal maize, with the most increased fraction being the zein protein, which was shown to contain no lysine soon after the long-term selection program initiated. Therefore, IHP is of little value for feeding humans and monogastric animals. Although high-lysine lines of non-vitreous mutants were based on reduced zeins, the kernel soft texture precluded their practical use. Kernel hardness in opaque 2 (o2) could be restored in quality protein maize (QPM) with quantitative trait loci called o2 modifiers (Mo2s), but those did not increase total protein levels.

**Methods:**

The most predominant zeins are the 22- and 19-kDa α-zeins. To achieve a combination of desired traits, we used RNA interference (RNAi) against both α-zeins in IHP and evaluated the silencing effect by SDS-PAGE. Total protein, amino acid composition and kernel texture were analyzed.

**Conclusions:**

The α-zeins were dramatically reduced, but the high total seed protein level remained unchanged by complementary increase of non-zein proteins. Moreover, the residual zein levels still allowed for a vitreous hard seed. Such dramatic rebalancing of the nitrogen sink could have a major impact in world food supply.

## Introduction

Maize (*Zea mays*), commonly known as corn, produces the highest yield among the major crops in the world, providing food for humans and feed for livestock. Although its yields are nearly four times higher than soybean (*Glycine max*), maize and other cereals are much less nutritious in terms of protein content and amino acid composition. Typical yellow dent maize contains 10% protein [1], of which the essential amino acid lysine is only around 2% [2], whereas soybean has 35% protein with sufficient levels of lysine. Therefore, maize meal is always supplemented with soybean in feed to meet the protein and lysine needs of livestock.

To improve protein concentration in maize, a well-known long-term selection-experiment was initiated in 1896 by C. G. Hopkins at the University of Illinois [3] and has lasted for more than a century [4], [5], [6]. At first, the highest- and lowest-scoring ears for protein from the open-pollinated variety Burr's White formed the parents for Illinois High Protein Strain (IHP) and Illinois Low Protein Strain (ILP), respectively. The reverse selection was initiated when 47 recurrent cycles of selecting high protein and low protein were finished, yielding another two strains, Reverse High protein (IRHP) and Reverse Low Protein (IRLP). Introgressed QTLs are capable of raising the protein concentration in IHP more than twice that in normal maize, with the most increased fraction being the alcohol-soluble proteins or prolamins. Seed storage proteins are classified into albumins, globulins, glutelins, and prolamins based on their solubilities in different solvents [7]. The major storage proteins in maize are prolamins, known as zeins, amounting to more than 60% of total protein. However, soon after the initiation of the Illinois long-term selection experiment in 1914, zein was shown to contain no lysine [8]. Lysine deficiency proved to cause the malnutrition disease pellagra when maize was consumed as the sole protein source. In nature, proteins are made of 20 kinds of L-amino acids, several of which, like lysine and tryptophan, cannot be synthesized by human beings and are called essential amino acids. If proteins are balanced, lysine content is supposed to be 5%. Because more than 60% of the zeins are almost lysine-free, the overall lysine in maize seed is reduced to a level as low as 2%. Therefore, despite its higher protein content, IHP is of little value for feeding monogastric animals. Indeed, the lysine content of total protein is even lower than that in normal maize because of the proportion of increased zeins.

This finding triggered a search for maize mutants with lower zein content and resulted in the discovery of high-lysine maize in the 1960s. The non-vitreous maize mutant, *opaque2* (*o2*) can double the lysine level [2]. Indeed, in the *o2* mutant, the amount of zeins is reduced by more than 60%, but the total protein level is balanced by an increase of non-zeins [9]. Unfortunately, non-vitreous *o2* kernels are soft and vulnerable to disease, so its initial commercialization was unsuccessful. Therefore, breeders at CYMMIT introduced QTLs, called *o2* modifiers, to select vitreous kernels to overcome the inferior properties of *o2* maize lines. The resulting modified maize is called quality protein maize (QPM) [10], [11]. As QPM is vitreous and high-lysine, it has been introduced in many developing countries in South America, Africa, and Asia [12]. Today, it is grown on over 10 million acres in 23 countries. However, conversion of QPM into local germplasms to develop hybrid maize is laborious. Moreover, once wildtype pollen blows onto the QPM ears, progeny lose their high lysine quality immediately. Recently, a new simpler QPM selection system, in which the dominant RNA interference (RNAi) against the 22- and 19-α zeins was coupled with the visible GFP marker, was developed to facilitate conversion of local germplasm [13]. Although QPM has more balanced amino acid composition than normal maize, its total protein level is much lower than that of soybeans. It seems that the three critical traits of valuable maize germplasm, high protein, high lysine, and hard endosperm remained an ever-lasting breeding challenge that could not be overcome all at once.

Still, comparing zein and non-zein fractions of the four Illinois Protein strains with inbred line B73, we found that IHP had the highest absolute lysine level (lysine_ab_) in total maize meal, but the lowest relative level of lysine (lysine_rel_) in total protein. The latter, however, determines whether the protein is balanced in amino acid composition from a nutritional point of view. To test whether mere reduction of alpha zeins in IHP would balance amino acid composition, we introduced into IHP an RNAi construct against both the 22- and 19-α zeins [13]. We found that reduction of zeins did not result in soft kernels or a reduction of total protein. The resulting seeds still contained 25% total protein, as high as the parent IHP line, and a lysine level that increased to 3.7%. Interestingly, because the RNAi construct did not reduce alpha zeins completely, the residual amount was sufficient to maintain a vitreous hard endosperm. Therefore, we show that it is possible to generate dominant vitreous, high-protein, high-lysine maize, an outcome that had not been achieved by conventional breeding in over a century. We suggest that such maize could reduce food cost worldwide significantly.

## Results and Discussion

### Protein and Amino Acid Composition in Illinois Protein Strains

**Figure 1:**
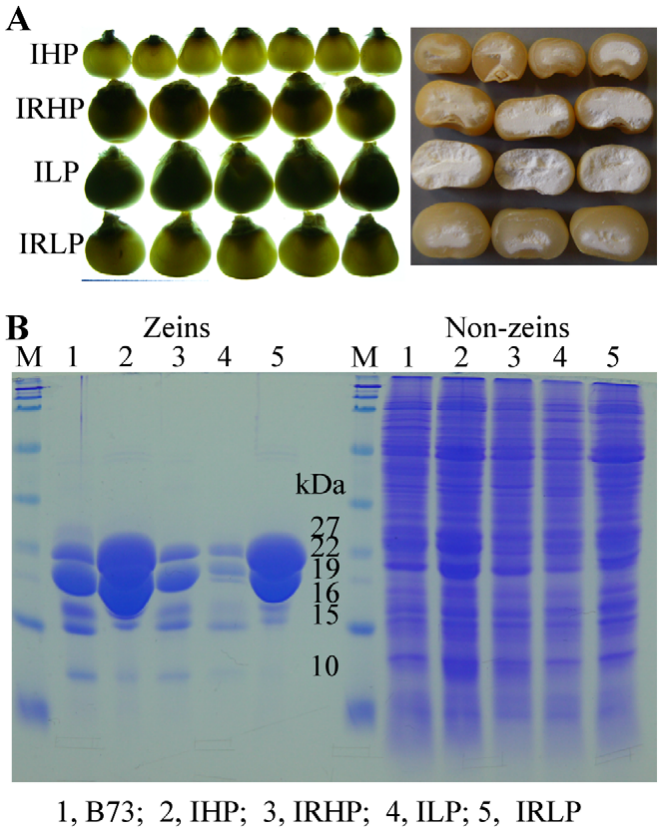
Kernel phenotype and protein accumulation pattern of Illinois Protein Strains. (A) Kernel translucency and vitreousness of IHP, IRHP, ILP and IRLP. (B) Zein and non-zein accumulation pattern of B73, IHP, IRHP, ILP and IRLP analyzed by 15% SDS-PAGE. Protein from 500 µg of maize flour was loaded in each lane. M, protein markers from top to bottom being 250, 150, 100, 75, 50, 37, 25, 20, 15 and 10 kDa. The size of each zein band is indicated with numbers in the “kDa” column.

The vitreousness of IRHP is between that of IHP, IRLP and ILP (Fig. 1A). IHP and IRLP kernels are translucent and vitreous, whereas ILP kernels are opaque and starchy. To correlate vitreousness with protein content, zeins and non-zeins were fractionated by different extraction solvents and separated electrophoretically by SDS-PAGE (Fig. 1A). Consistent with their kernel phenotypes, IHP and IRLP accumulated the highest and second highest amount of zeins, respectively, with the most prominent bands being the 22- and 19-kDa α-zeins. They are followed by B73, IRHP and ILP in respect to zein accumulation. Interestingly, non-zein protein accumulation from highest to lowest is also in the same order: IHP>IRLP>B73>IRHP>ILP by careful pair-wise comparisons. Apparently, the long-term breeding-program accumulated QTLs in IHP that not only increased zeins but also non-zein proteins, suggesting a more general mechanism of controlling total seed protein content. Because lysine_ab_ relates to the proportion of non-zeins in seeds, the contribution of lysine_ab_ is expected to be proportional to non-zeins. Indeed, IHP contains the highest level of lysine with 0.36%, while ILP the lowest with only 0.2% (Table 1). On the contrary, the relative level of lysine (lysine_rel_), which is of nutritional value, is calculated as a percentage of lysine in total protein, directly proportional to the percentage non-zeins in total protein. Therefore, it was not unexpected that ILP have the highest lysine_(rel)_ (4.35%) level and IHP the lowest (1.47%) (Table 1). However, ILP and IRHP are not suitable choices from a commercial point of view because their total protein concentration is too low, requiring soybean supplementation (Table 1), definitely compromising their nutritional potential. Furthermore, ILP and IRHP are opaque and semi-opaque, indicators for soft kernel texture (Fig. 1A).

**Table 1:** Protein and amino acid composition analysis of four Illinois Protein Strains (IHP, IRHP, ILP and IRLP) and B73.

Amino acids	IHP		IRHP		ILP		IRLP		B73	
	AA_ab_	AA_rel_	AA_ab_	AA_rel_	AA_ab_	AA_rel_	AA_ab_	AA_rel_	AA_ab_	AA_rel_
Lysine	0.36%	####	0.28%	4.12%	0.20%	4.35%	0.32%	2.05%	0.31%	2.54%
Phenylalanine	1.24%	####	0.34%	5.00%	0.18%	3.91%	0.90%	5.77%	0.63%	5.16%
Leucine	3.85%	####	0.75%	11.03%	0.41%	8.91%	2.35%	15.06%	1.65%	13.52%
Isoleucine	0.50%	####	0.21%	3.09%	0.11%	2.39%	0.50%	3.21%	0.40%	3.28%
Threonine	0.65%	####	0.27%	3.97%	0.17%	3.70%	0.50%	3.21%	0.41%	3.36%
Valine	0.73%	####	0.32%	4.71%	0.16%	3.48%	0.59%	3.78%	0.48%	3.93%
Histidine	0.55%	####	0.22%	3.24%	0.12%	2.61%	0.41%	2.63%	0.37%	3.03%
Arginine	0.70%	####	0.32%	4.71%	0.22%	4.78%	0.59%	3.78%	0.54%	4.43%
Glycine	0.60%	####	0.34%	5.00%	0.22%	4.78%	0.46%	2.95%	0.49%	4.02%
Aspartic acid	1.63%	####	0.46%	6.76%	0.37%	8.04%	0.95%	6.09%	0.74%	6.07%
Serine	1.28%	####	0.35%	5.15%	0.22%	4.78%	0.79%	5.06%	0.60%	4.92%
Glutamic acid	5.23%	####	1.10%	16.18%	0.65%	#####	3.04%	19.49%	2.26%	18.52%
Proline	2.08%	####	0.59%	8.68%	0.34%	7.39%	1.33%	8.53%	1.15%	9.43%
Hydroxyproline	0.02%	####	0.03%	0.44%	0.02%	0.43%	0.04%	0.26%	0.03%	0.25%
Alanine	2.26%	####	0.53%	7.79%	0.33%	7.17%	1.41%	9.04%	0.94%	7.70%
Tyrosine	0.93%	####	0.24%	3.53%	0.12%	2.61%	0.53%	3.40%	0.45%	3.69%
Total protein	24.50%		6.80%		4.60%		15.60%		12.20%	

AA_ab_, absolute level of amino acid calculated by percentage of AA in total cornmeal; AA_rel_, relative level of amino acid calculated by percentage of AA in total protein.

### RNA Interference of Zeins in Illinois Reverse Low Protein (IRLP)

Traits of high protein, high lysine, and hard endosperm seem to exclude each other in maize. While higher amounts of zeins increase kernel vitreousness (Fig. 1A), they also decrease the lysine_rel_ at the same time, because lysine_rel_ is inversely proportional to the percentage of zeins of total protein. QPM overcame two of the three traits, producing high lysine and hard kernel properties. However, current QPM germplasms developed at CYMMIT and in South Africa, contain just around 9% protein, which is not comparable to IHP or soybeans [12].

**Figure 2:**
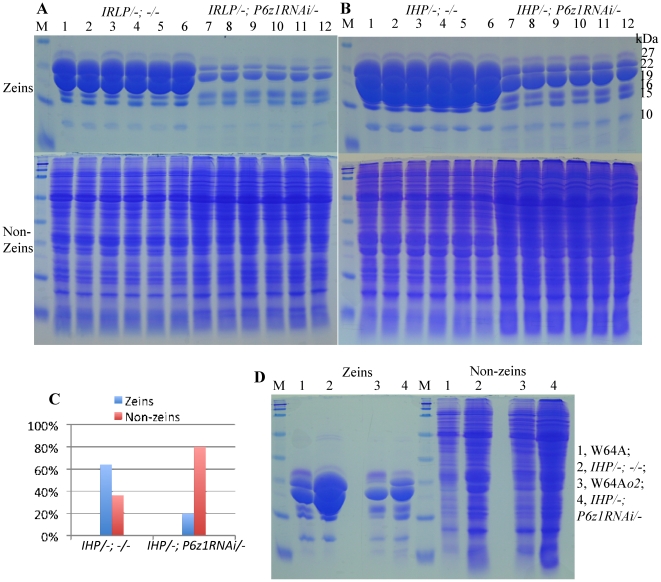
Rebalancing of zein and non-zein ratio in IRLP and IHP. Zeins and non-zeins extracted from six “non-green” and “green” kernels (lanes 1–6 and 7–12, respectively), each from IRLP x *P6z1RNAi/-* (A) and IHP x *P6z1RNAi/-* (B). They were scored for GFP with fluorescent light microscope and analyzed individually by 15% SDS-PAGE. (C) Comparison of zein and non-zein ratio in total protein between *IHP/-*; *-/-* and *IHP/-*; *P6z1RNAi/-*. (D) Comparison of zein and non-zein accumulation patterns in W64A, W64A*o2*, *IHP/-*; *-/-* and *IHP/-*; *P6z1RNAi/-*. The total protein loaded in each lane was equal to 500 µg of maize flour. M, protein markers in the top panels of (A) and (B) from top to bottom being 37, 25, 20, 15 and 10 kDa, and in (D) and the bottom panels of (A) and (B) being 250, 150, 100, 75, 50, 37, 25, 20, 15 and 10 kDa. The size for each zein band is marked with numbers in the “kDa” column.

**Figure 3:**
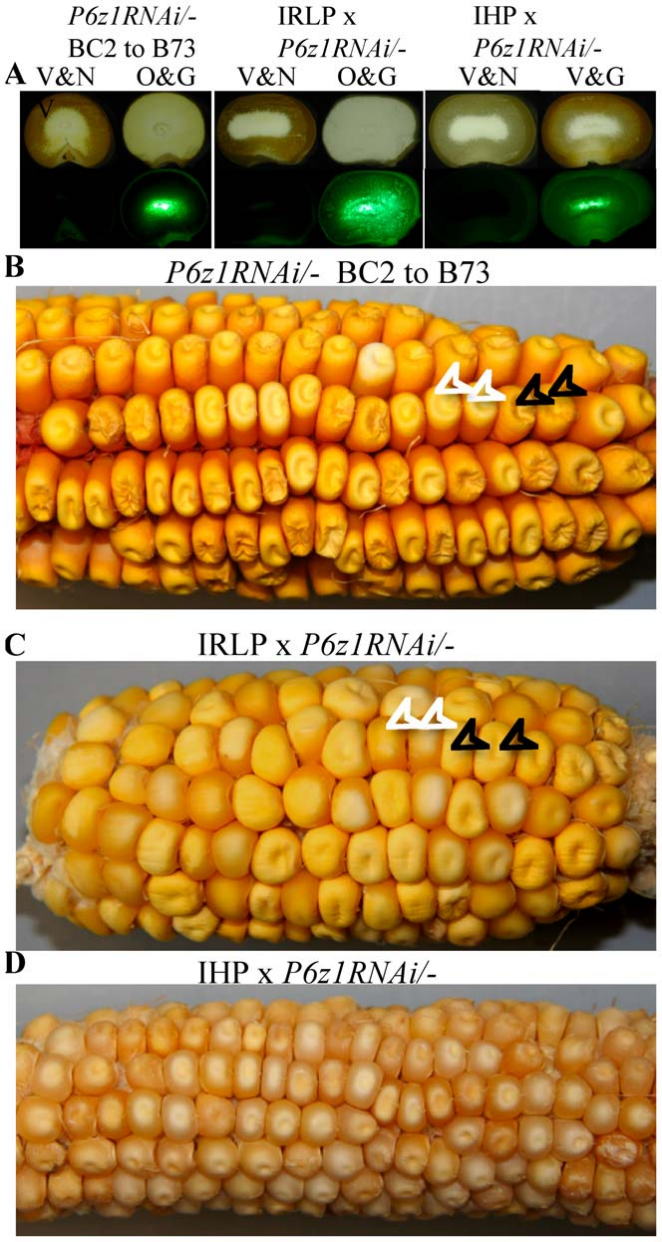
Ear phenotype of *P6z1RNAi* backcrossed to B73, IRLP and IHP. (A) Kernels were cut in half for observation under a natural light (upper panel) and fluorescence dissection microscope (bottom panel). Opaque phenotype in kernels from the ear with *P6z1RNAi* backcrossed to B73 for two generations and IRLP x *P6z1RNAi/-* is linked to the expression of GFP. In IHP x *P6z1RNAi/-*, the non-green and green kernels are both vitreous. V&N, vitreous and non-green; O&G, opaque and green; V&G, vitreous and green; The ear with *P6z1RNAi* backcrossed to B73 for two generations (B) and IRLP x *P6z1RNAi/-* (C) show 1∶1 ratio of vitreous and opaque segregation. Two vitreous and opaque kernels in each ear are indicated by white and black arrows, respectively. (D) In IHP x *P6z1RNAi/-*, kernels were uniformly vitreous.

If we were to increase lysine in IRLP and IHP with a reduction of alpha zeins through RNAi, we would expect to obtain a non-vitreous soft seed as we have previously shown with normal maize [14]. On the other hand, we also found that the QPM QTLs were dominant over non-vitreousness caused by an alpha zein RNAi. Perhaps, the QTLs of IRLP and IHP would also be dominant over an alpha zein RNAi. Therefore, we pollinated IRLP and IHP with pollen from B73 containing the transgene *P6z1RNAi* because progenies should exhibit high protein traits only when IRLP and IHP were used as female parent [15]. In the construct P6z1RNAi, the 22- and 19-kDa α-zein RNAi was coupled with the visible GFP marker, therefore parental seeds were opaque and “green” in normal maize lines [13]. The progenies inheriting the construct were easy to score under a green fluorescent dissection microscope. Six “non-green” and “green” kernels sorted from IRLP x *P6z1RNAi/-* and IHP x *P6z1RNAi/-* were extracted for zeins and non-zeins individually (see Methods) and the proteins were also separated with SDS-PAGE (Fig. 2A and 2B). As expected, the accumulation of α-zeins in the six “green” progenies from IRLP x *P6z1RNAi/-* was dramatically reduced compared to the six “non-green” progenies. Although protein levels are already high for maize seeds, a rebalancing of protein accumulation still occurred as the accumulation of non-zeins in the six “green” progenies was significantly increased (Fig. 2A). As expected from the previous work, kernel opacity was linked to the expression of GFP (Fig. 3A) and the ear exhibited 1∶1 ratio of vitreous and opaque segregation (Fig. 3C). The same was the case, when the line with *P6z1RNAi* was backcrossed to B73 for two generations (Fig. 3B). Therefore, the QTLs of IRLP were not dominant over *P6z1RNAi* as hoped for.

### RNA Interference of Zeins in Illinois High Protein (IHP)

The result for IHP was strikingly different. Similar to IRLP x *P6z1RNAi/-*, six “green” progenies from IHP x *P6z1RNAi/-* had dramatic decrease in α-zein synthesis compared to their “non-green” counterparts (Fig. 2B). However, while IHP and the progenies with genotype *IHP/-; -/-* accumulated higher levels of α-zeins than IRLP and *IRLP/-; -/-*, respectively (Figs. 1B and 2A–B), there was still significant more α-zeins in *IHP/-; P6z1RNAi/-* than in *IRLP/-; P6z1RNAi/-*. Like *IRLP/-; P6z1RNAi/-*, the non-zein fraction in *IHP/-; P6z1RNAi/-* was dramatically enhanced compared to the progenies with genotype *IHP/-; -/-*. Moreover, the ratios of zeins and non-zeins in total protein of *IHP/-; -/-* (64% and 36%, respectively) were shifted to 20% and 80% in *IHP/-; P6z1RNAi/-* because of RNA interference (Fig. 2C). When the accumulations of zeins and non-zeins were compared between W64A, *IHP/-; -/-*, W64A*o2* and *IHP/-; P6z1RNAi/-*, the reduced level of zeins in *IHP/-; P6z1RNAi/-* was still as high as that in normal W64A (Fig. 2D), although it dropped to two third of zeins compared to genotype *IHP/-; -/-* (Fig. 2B and 2C).

**Figure 4:**
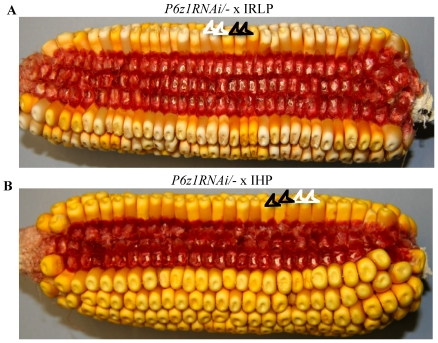
Ear phenotype of *P6z1RNAi/-* x IRLP (A) and *P6z1RNAi/-* x IHP (B). Vitreous and opaque kernels segregate as 1∶1 ratio in both crosses. Two representative kernels for each phenotype are indicated by white and black arrows, respectively.

The residual amount of alpha zeins is sufficient to produce vitreous kernels in the genotype *IHP/-; P6z1RNAi/-*. GFP positive kernels of *IHP/-; P6z1RNAi/-* are, indeed, vitreous (Fig. 3A) and no segregation is seen in the *IHP/-; P6z1RNAi/-* ear (Fig. 3D). When the transgenic line *P6z1RNAi*, backcrossed twice to B73, was pollinated either by IHP or IRLP, the resulting ears segregated 1∶1 ratio of vitreous and opaque kernels (Fig. 4A and 4B), confirming that expression of the QTLs of high protein requires maternal transmission. In summary, IHP QTLs were dominant over *P6z1RNAi*, but the QTLs of IRLP were recessive, because kernels of *IHP/-; P6z1RNAi/-* were vitreous and those with genotype *IRLP/-; P6z1RNAi/-* were opaque. Furthermore, QTLs affect total protein accumulation rather than zein synthesis alone (Fig. 2B and Table 2).

**Table 2:** Protein and amino acid composition analysis of seeds with genotype IHP/-; -/-, IHP/-; P6z1RNAi/-, IRLP/-; -/-, IRLP/-; P6z1RNAi/- and W64Ao2.

Amino acids	*IHP/-; -/-*		*IHP/-; P6z1RNAi/-*		*IRLP/-; -/-*		*IRLP/-; P6z1RNAi/-*		W64A*o2*	
	AA_ab_	AA_rel_	AA_ab_	AA_rel_	AA_ab_	AA_rel_	AA_ab_	AA_rel_	AA_ab_	AA_rel_
Lysine	0.46%	1.73%	0.91%	3.68%	0.37%	2.52%	0.58%	4.06%	0.48%	3.93%
Phenylalanine	1.44%	5.41%	0.99%	4.01%	0.76%	5.17%	0.60%	4.20%	0.54%	4.43%
Leucine	4.31%	16.20%	2.18%	8.83%	2.03%	13.81%	1.19%	8.32%	1.06%	8.69%
Isoleucine	0.65%	2.44%	0.55%	2.23%	0.45%	3.06%	0.43%	3.01%	0.27%	2.21%
Threonine	0.75%	2.82%	0.83%	3.36%	0.49%	3.33%	0.54%	3.78%	0.44%	3.61%
Valine	0.86%	3.23%	0.82%	3.32%	0.60%	4.08%	0.64%	4.48%	0.41%	3.36%
Histidine	0.78%	2.93%	0.80%	3.24%	0.43%	2.93%	0.45%	3.15%	0.43%	3.52%
Arginine	0.85%	3.20%	1.27%	5.14%	0.59%	4.01%	0.80%	5.59%	0.67%	5.49%
Glycine	0.67%	2.52%	1.02%	4.13%	0.41%	2.79%	0.58%	4.06%	0.58%	4.75%
Aspartic acid	1.75%	6.58%	3.46%	14.01%	1.17%	7.96%	1.49%	10.42%	1.19%	9.75%
Serine	1.38%	5.19%	1.07%	4.33%	0.73%	4.97%	0.68%	4.76%	0.61%	5.00%
Glutamic acid	5.71%	21.47%	4.20%	17.00%	3.10%	21.09%	2.71%	18.95%	2.21%	18.11%
Proline	2.31%	8.68%	1.65%	6.68%	1.19%	8.10%	1.09%	7.62%	0.98%	8.03%
Hydroxyproline	0.02%	0.08%	0.02%	0.08%	0.02%	0.14%	0.04%	0.28%	0.04%	0.33%
Alanine	2.37%	8.91%	1.67%	6.76%	1.38%	9.39%	1.10%	7.69%	1.02%	8.36%
Tyrosine	1.04%	3.91%	0.76%	3.08%	0.52%	3.54%	0.44%	3.08%	0.38%	3.11%
Total protein	26.60%		24.70%		14.70%		14.30%		12.20%	

AA_ab_, absolute level of amino acid calculated by percentage of AA in total cornmeal; AA_rel_, relative level of amino acid calculated by percentage of AA in total protein.

### Selection of High Lysine-rich Protein with Hard Endosperm

The aspect that the QTLs in IHP could enhance both zeins and non-zein proteins is critical, because lysine_rel_, appears to be not affected by the absolute amount of zeins in maize meal, but by the ratio of zeins and non-zeins. Although *IHP/-; P6z1RNAi/-* still produces higher amount of zeins despite RNA interference compared to W64A*o2*, the critical difference is that its non-zein fraction is significantly higher than in W64A*o2* (Fig. 2D). From a perspective of a sink-source relationship of amino acids, QTLs would be dominant and the seeds retain the high protein property. Furthermore, the high non-zein protein content should also lift lysine levels in the seed. Indeed, whereas the total protein level in *IHP/-; P6z1RNAi/-* (24.7%) was high as in IHP (24.5%) and *IHP/-; -/-* (26.60%), the lysine_rel_ in *IHP/-; P6z1RNAi/-* was 3.7%, as balanced as in W64*o2* (Table 2). Although the lysine_rel_ in *IRLP/-; P6z1RNAi/-* was above 4%, the kernels were opaque and its practical application would then require layering the QPM QTLs on top of the high protein QTLs, constituting a major challenge to breeders. Therefore, these results show that the RNAi construct *P6z1RNAi* can now be used to identify QTLs for high protein, high lysine, and a vitreous hard endosperm, thereby providing superior maize lines that could have a high impact on the cost of food supply worldwide. Because these QTLs have to be combined with other agronomic traits like yield, they will have to be back-crossed to elite germplasm for practical application. Indeed, one could use an accelerated breeding strategy as recently proposed for the introgression of QPM QTLs into local germplasm [13].

## Methods

### Genetic Stocks

The *P6z1RNAi* transgenic plant has been described previously [13]. The *P6z1RNAi* transgenic plant was then backcrossed to B73 for two generations (Fig. 3A), which consistently showed 1∶1 ratio of vitreous (non-green) and opaque (green) seeds segregating in each generation.

The four Illinois Protein Strains (IHP, IRHP, ILP and IRLP) were obtained from Dr. Stephen Moose of the University of Illinois. B73, W64A and W64*o2* were from our own stocks.

### Total Zein and Non-zein Protein Extraction, Protein and Amino Acid Composition Analysis

For zein extraction, the dry kernels were wrapped individually in two layers of thick aluminium foil and crushed into fine flour by a heavy hammer. In Figure 1B, at least three kernels for each line were wrapped. For segregation analysis in Figure 2A and 2B, kernels were ground individually. Only 50 mg of flour was transferred to a 2 ml Eppendorf tube, then mixed and vortexed with 400 µl of 70% ethanol/2% 2-mercaptoethanol (v/v), then kept on the bench at room temperature overnight; the mixture was centrifuged at 13,000 rpm in a benchtop microfuge for 10 min, then 100 µl of the supernatant liquid was transferred to a new tube; 10 µl of 10% SDS was added to the extract, the mixture was dried by vacuum and resuspended in 100 µl of distilled water.

For non-zein extraction, the supernatant from above was discarded. Solids remaining in the tube were resuspended with zein extraction buffer to completely remove the zeins from other proteins. This step was repeated for 3 times. At last, the residual solids were suspended in 400 µl of non-zein extraction buffer (12.5 mM sodium borate, 5% SDS and 2% 2-mercaptoethanol (vol/vol)). The mixture was kept at 37°C for two hours and vortexed several times in this period. The mixture was centrifuged at 13,000 rpm for 10 min, and then 100 µl of the non-zein supernatant was transferred to a new tube. 4 µl (equal to 500 µg of floury) of each sample was analyzed with 15% SDS-PAGE gel, run at 200 Voltage for 35 min. The resulting gel was stained with Coomassie buffer.

The concentrations of zein and non-zein proteins in Figure 2C were measured with Bradford Protein Assay Kit (Bio-Rad).

About 20 g of mature seeds were ground to fine flour. The protein and amino acid composition analysis was conducted by the New Jersey Feed Laboratory, Inc., Trenton, NJ, USA.

### Incandescent and Fluorescent Light Dissection Microscopy

Kernels were truncated and scoped under incandescent and fluorescent light dissection microscopes, respectively (WILD M3 and Leica MZ16 F).
