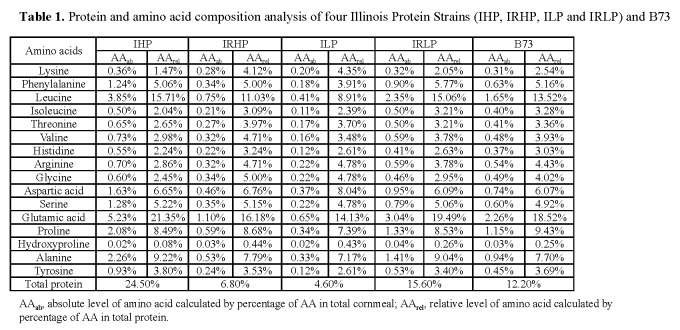# Correction: RNA Interference Can Rebalance the Nitrogen Sink of Maize Seeds without Losing Hard Endosperm

**DOI:** 10.1371/annotation/06346ecc-81e4-4cec-8923-0551781a416a

**Published:** 2012-06-08

**Authors:** Yongrui Wu, Joachim Messing

There was an error in Table 1. The correct Table 1 can be viewed here: 

**Figure:**